# Clinical Use of Aspirin in Treatment and Prevention of Cardiovascular Disease

**DOI:** 10.1155/2012/245037

**Published:** 2011-11-24

**Authors:** Yuxiang Dai, Junbo Ge

**Affiliations:** Shanghai Cardiovascular Institute and Department of Cardiology, Zhongshan Hospital, Fudan University, Shanghai 200032, China

## Abstract

Cardiovascular disease (CVD), principally heart disease and stroke, is the leading cause of death for both males and females in developed countries. Aspirin is the most widely used and tested antiplatelet drug in CVD, and it is proven to be the cornerstone of antiplatelet therapy in treatment and prevention of CVD in clinical trials in various populations. In acute coronary syndrome, thrombotic stroke, and Kawasaki's disease, acute use of aspirin can decrease mortality and recurrence of cardiovascular events. As secondary prevention, aspirin is believed to be effective in acute coronary syndrome, stable angina, revascularization, stroke, TIA, and atrial fibrillation. Aspirin may also be used for patients with a high risk of future CVD for primary prevention, but the balance between benefits and the possibility of side effects must be considered.

## 1. Introduction

Cardiovascular disease (CVD) continues to be the leading clinical and public health problem in developed countries and increasingly so throughout the world. Heart disease and stroke are the two main manifestations associated with CVD. The World Health Organization estimates that CVD will be the leading cause of death and disability worldwide by the year 2020 [1].

Millions of patients worldwide take low-dose aspirin on a daily basis for the treatment and prevention of CVD. By far, aspirin is the most widely tested antiplatelet drug in randomized trials of treatment and prevention of CVD [2]. Despite being one of the most widely used drugs in the 20th century, the benefits of aspirin in CVD have only relatively recently been recognized. This paper aims to provide clinical practice with a review of the evidence related to the use of aspirin for the treatment and prevention of cardiovascular events.

## 2. Mechanism of Action

Aspirin's mechanism of action involves inhibition of platelet activation and aggregation, which was first described in 1971 by British pharmacologist John Vane [3]. He demonstrated that the main mechanism of action was the irreversible inhibition of the platelet-dependent enzyme cyclooxygenase (COX), thereby preventing the synthesis of prostaglandins. Subsequent researchers identified two COX isoenzymes, COX-1 and COX-2 [4, 5]. In platelets, the COX-1 enzyme produces thromboxane A2, a powerful promoter of platelet aggregation. Thus, aspirin, by irreversibly inactivating COX-1, thereby blocking the generation of thromboxane A2, derives a potential antiplatelet effect [6]. 

Platelet activation and aggregation with subsequent activation of the clotting cascade play critical roles in the onset of acute occlusive vascular events, such as MI and occlusive cerebrovascular accident (CVA) [7]. Because platelets do not have nucleus and thus cannot regenerate COX, they become an excellent target for antithrombotic therapy, while aspirin shows both immediate and long-term effects on platelets [8].

Other mechanisms of aspirin in CVD may also work. Aspirin blocks the formation of COX-dependent vasoconstrictors, which contribute to endothelial dysfunction in atherosclerosis [9]. Thus, improvement of endothelial dysfunction with aspirin may improve vasodilation, reduce thrombosis, and inhibit progression of atherosclerosis. Furthermore, aspirin reduces the inflammatory response in patients with coronary artery disease [10] and may inhibit the progression of atherosclerosis by protecting low-density lipoprotein from oxidation [11].

## 3. Treatment in Cardiovascular Disease

### 3.1. Therapy for Acute Coronary Syndrome

Convincing data support the use of aspirin in the acute treatment of acute coronary syndrome (ACS), including ST-elevation myocardial infarction (STEMI), non-ST-elevation myocardial infarction (NSTEMI) and unstable angina pectoris (UA) [12–14]. For ACS patients, the current American Heart Association/American College of Cardiology (AHA/ACC) guidelines recommend that aspirin should be administered as soon as possible with an initial loading dose of 162–325 mg and continued indefinitely with a dose of 75–162 mg daily [15, 16]. In the second International Study of Infarct Survival (ISIS-2) study, the use of aspirin (162 mg chewed, to ensure rapid therapeutic blood levels) was associated with a 23% reduction of vascular mortality rate in MI patients and close to a 50% reduction of nonfatal reinfarction or stroke, with benefits seen in both men and women [12]. In UA and USTEMI patients, aspirin has been shown to reduce the risk of fatal or nonfatal MI by 50–70% during the acute phase and by 50–60% at 3 months to 3 years [13, 14]. 

The highest benefit of aspirin was seen in those undergoing coronary angioplasty, with a 53% (*P* < 0.0002) reduction in MI, stroke, or vascular deaths [17]. In percutaneous coronary intervention (PCI), the use of aspirin significantly reduces abrupt closure after balloon angioplasty and significantly reduces stent thrombosis rates [18]. 

### 3.2. Therapy for Kawasaki's Disease

Kawasaki's disease, which is a kind of acute vasculitis, occurs most commonly in children and in 15 to 25% of untreated cases results in the development of coronary artery aneurysms [19]. In the consensus guidelines from the Seventh American College of Chest Physicians (ACCP) Conference on Antithrombotic and Thrombolytic Therapy, high-dose aspirin (80–100 mg/kg/day) is recommended during the acute phase of the illness for its antiinflammatory effects, followed by low-dose aspirin (3–5 mg/kg/day) for its antiplatelet effect for 7 weeks or longer, maintaining it until the patient shows no evidence of coronary changes. In children with coronary aneurysms, long-term anticoagulation with warfarin and low-dose aspirin is recommended [20]. 

### 3.3. Therapy for Thromboembolic Stroke

With regard to stroke, the International Stroke Trial (IST) [21] and the Chinese Acute Stroke Trial (CAST) [22] together enrolled more than 40,000 patients admitted to hospital within 48 hours of the onset of stroke symptoms, who were randomized within 48 hours of the onset of symptoms to 2 to 4 weeks of daily aspirin therapy (300 mg and 160 mg, resp.) or placebo. Results from both trials suggest that aspirin therapy decreased the risk of recurrent stroke and death without significantly increasing the risk of hemorrhagic stroke [21, 22]. These results are consistent with biochemical evidence of episodic platelet activation during the first 48 h after the onset of symptoms of an acute ischemic stroke and with suppression of in vivo TXA2 biosynthesis in patients receiving low-dose aspirin in this setting.

## 4. Secondary Prevention

Secondary prevention refers to the use of aspirin to prevent cardiovascular and cerebrovascular events in patients who have already experienced such an event or who have a high risk of an event. Long-term aspirin therapy reduces the yearly risk of serious vascular events (nonfatal myocardial infarction, nonfatal stroke, or vascular death), which corresponds to an absolute reduction of nonfatal events and to a smaller, but still definite, reduction in vascular death. Against these benefits, the absolute increase in major gastrointestinal or other major extracranial bleeds is relatively smaller. Hence, for secondary prevention, the benefits of aspirin therapy substantially exceed the risks, and aspirin is recommended as secondary prevention in conjunction with lifestyle change and stopping smoking to reduce an individual's overall risk of further cardiovascular events. 

The Antithrombotic Trialists' (ATT) Collaboration performed a meta-analysis in 2002, which examined 287 randomized studies with 135000 high-risk patients in comparisons of antiplatelet therapy (predominantly aspirin) versus control and 77000 in comparisons of different antiplatelet regimens [17]. The results showed that among these high-risk patients, including acute MI, acute stroke, previous stroke or transient ischemic attack (TIA), peripheral arterial disease, atrial fibrillation, antiplatelet therapy reduced the combined outcome of any serious vascular event by about 25%, reduced nonfatal myocardial infarction by about 33%, reduced nonfatal stroke by about 25%, and reduced vascular mortality by about 17%. In each of the high-risk categories, the absolute benefits outweighed the absolute risks of major extracranial bleeding.

For the choice of aspirin dosage, this analysis showed that COX is virtually completely inhibited in platelets, producing an antithrombotic effect, within a few days of beginning 75 mg aspirin daily. It was indicated that high doses of 500–1500 mg aspirin daily (which are more gastrotoxic48) were no more effective than medium doses of 160–325 mg/day or low doses of 75–150 mg/day. Low-dose aspirin (75–150 mg daily) is an effective antiplatelet regimen for long-term use, and the effects of doses lower than 75 mg daily were less certain. In clinical acute settings requiring an immediate antithrombotic effect (such as acute myocardial infarction, acute ischaemic stroke, unstable angina), an initial loading dose of about 150–300 mg aspirin should probably be given [17]. 

More recently, ATT Collaboration conducted another meta-analysis involving 16 secondary prevention trials (17 000 individuals at high average risk, 43 000 person-years, 3306 serious vascular events) that compared long-term aspirin versus control. This analysis showed that aspirin allocation yielded a greater absolute reduction in serious vascular events (6.7% versus 8.2% per year, *P* < 0.0001), with a nonsignificant increase in haemorrhagic stroke but reductions of about 20% in total stroke (2.08% versus 2.54% per year, *P* = 0.002) and in coronary events (4.3% versus 5.3% per year, *P* < 0.0001) [23].

Aspirin (or another oral antiplatelet drug) is protective in most types of patient at increased risk of occlusive vascular events, including those with an acute myocardial infarction or ischaemic stroke, unstable or stable angina, previous myocardial infarction, stroke or cerebral ischaemia, peripheral arterial disease, or atrial fibrillation.

### 4.1. Secondary Prevention for Acute Coronary Syndromes

The benefit of aspirin therapy for preventing cardiovascular events in patients with ACS (STEMI, USTEMI, UP) has been definitively demonstrated in several trials [13, 14, 24, 25]. The previous meta-analysis by the ATT Collaboration [17] reviewed 18788 patients with a history of MI from the 12 most important randomized clinical trials of aspirin and showed that aspirin therapy reduced the relative risk of nonfatal MI by 28% (*P* < 0.0001), vascular death by 15% (*P* < 0.0006), and overall mortality by 11% (*P* = 0.02). The daily dosage of 80–325 mg appears to be effective in reducing the risk of cardiovascular events.

The 2007 ACC/AHA guidelines for the management of patients with unstable angina and non-ST-segment elevation MI recommend initiating daily aspirin therapy with at least 162 mg as soon as possible after clinical presentation, with 75–325 mg daily indefinitely thereafter [15]. The 2004 ACC/AHA guidelines for the management of patients with ST-segment elevation MI are similar but recommend 75–162 mg daily as maintenance therapy after ST-segment elevation MI. Aspirin therapy is considered a class I recommendation (evidence supports that treatment is useful and effective) for all acute coronary syndromes [16]. The initial dose of aspirin should be chewed and then swallowed during acute coronary syndromes to attain a rapid onset of action.

### 4.2. Secondary Prevention for Chronic Stable Angina

A subgroup analysis of the US Physicians' Health Study (PHS) of 333 men with chronic stable angina indicated that aspirin reduced the relative risk of acute MI by 87% (*P* < 0.001) [26]. The Swedish Angina Pectoris Aspirin Trial involved 2035 patients and found a 34% relative risk reduction in the occurrence of a first MI over a four-year follow-up period in patients receiving 75 mg of aspirin daily, compared with patients receiving placebo [27].

The 2002 ACC/AHA guidelines for chronic stable angina include a class IIa recommendation (the weight of evidence where opinion is in favor of usefulness and efficacy) for prophylactic aspirin therapy to prevent MI and death [28].

### 4.3. Secondary Prevention for Revascularization

Aspirin has been widely accepted as a cornerstone therapy in reducing ischemic complications of coronary revascularization with either coronary artery bypass graft surgery, balloon angioplasty, or stent implantation [29–31]. A number of studies have demonstrated the efficacy of aspirin in preventing thrombosis, a common event following revascularization [32–35].

Aspirin administered in the immediate postoperative period following bypass surgery decreases the rate of graft occlusion by approximately 50%, and continued therapy leads to further decreases [29, 34]. Use of aspirin before and after coronary intervention is essential in the prevention of thrombosis. Early trials indicated that, in patients undergoing PCI, aspirin reduced mortality, MI, urgent revascularization, or stent thrombosis both with and without thienopyridines [18, 36–38]. 

The 2004 ACC/AHA guidelines for coronary artery bypass graft surgery suggest daily aspirin therapy with 100–325 mg started within 24 hours after surgery [39]. The 2005 ACC/AHA guidelines for percutaneous coronary intervention recommend 75–325 mg of aspirin before the PCI procedure is performed in patients already taking daily chronic aspirin therapy, and 300–325 mg of aspirin at least 2 hours and preferably 24 hours before the PCI procedure is performed in patients not already taking daily chronic aspirin therapy [40]. After the PCI procedure, in patients with neither aspirin resistance, allergy, nor increased risk of bleeding, aspirin 162–325 mg daily should be given for at least 1 month after BMS implantation, 3 months after sirolimus-eluting stent implantation, and 6 months after paclitaxel-eluting stent implantation, after which daily chronic aspirin use should be continued indefinitely at a dose of 75 to 162 mg [40]. All of these recommendations belong to class I (evidence supports that treatment is useful and effective).

### 4.4. Secondary Prevention for Stroke and Transient Ischemic Attack

The previous meta-analysis by the ATT Collaboration involved 18270 patients with a history of stroke or transient ischaemic attack in 21 trials [17]. The result showed that antiplatelet therapy (mainly aspirin alone) for a mean duration of 29 months can significantly reduce the rate of major vascular events by 22%. Treating 1000 patients with a history of cerebrovascular disease for this duration will prevent about 36 vascular events, mostly nonfatal stroke recurrence (25 fewer per 1000 treated), and some nonfatal myocardial infarction (5 fewer per 1000).

### 4.5. Secondary Prevention for Atrial Fibrillation

The presence of atrial fibrillation (AF) gives rise to the development of atrial thrombus and consequently increases the risk of stroke among elderly people. Vitamin K antagonists, most notable among which is warfarin, significantly reduce the risk of stroke by almost two-thirds compared to placebo. Owing to the difficulties with using warfarin of its requirement for frequent monitoring of the INR and increased hemorrhagic risk with increased duration of therapy, aspirin has been considered a potential alternate [41–43].

Most of the evidence about the effects of aspirin therapy among patients with atrial fibrillation was provided by the European atrial fibrillation trial [44]. High-risk patients with a previous stroke or transient ischemic attack were randomized to aspirin or placebo (or oral anticoagulant, if eligible) in this trial. It was indicated that aspirin is a safe, though less effective, alternative when anticoagulation is contraindicated. Aspirin prevents 40 vascular events for every 1,000 treated patients. The previous meta-analysis by the ATT Collaboration [17] included 2770 patients with atrial fibrillation in four trials and found that there was a proportional reduction of 24% (9%) in serious vascular events. 

In patients with “lone AF,” who are under 65 years of age, not hypertensive, without evidence of cardiovascular disease and who have normal echocardiograms, the baseline stroke risk of this cohort is relatively low (approximately 0.5%/year). In this situation, aspirin alone is considered by most experts to be adequate [41–43].

## 5. Primary Prevention

For primary prevention, the balance between benefits and risks of aspirin use is less clear because the absolute benefits of aspirin are generally lower than those in secondary prevention. Current guidelines largely ignore any differences in bleeding risk and recommend that aspirin be used widely for primary prevention in those at moderately raised risk of coronary heart disease. It has also been suggested that, since age is a major determinant of the risk of coronary heart disease, daily aspirin should be started in all people above a specific age, either alone or in combination with other drugs.

To date, six completed randomized trials have evaluated the benefits and risks of low-dose aspirin for the primary prevention of cardiovascular disease. The British Male Doctors' Trial (BDT) [45] of 5139 male physicians and the US Physicians' Health Study (PHS) [26] of 22071 healthy male were completed during the late 1980s. The Thrombosis Prevention Trial (TPT) [46] of 5085 men and the Hypertension Optimal Treatment (HOT) [47] trial of 18790 (47% women) patients were completed in 1998. The Primary Prevention Project (PPP) [48] study of 4495 (58% women) patients and the Women's Health Study (WHS) [49] of 39876 healthy females were completed in the 2000s. In all these trials patients were randomized to aspirin and had follow-up durations ranging from 3.6 to 10.1 years. The PHS and BDT used aspirin regimens of 325 mg every other day and 500 mg/day, respectively, whereas the TPT and HOT used 75 mg/day of aspirin and the PPP and WHS used 100 mg/day of enteric-coated aspirin.

The Antithrombotic Trialists' (ATT) Collaboration undertook a meta-analysis in the 6 previous trials and found that, in the primary prevention trials, aspirin use yielded a 12% proportional reduction in serious vascular events (0.51% aspirin versus 0.57% control per year, *P* = 0.0001), due mainly to about 20% reduction in nonfatal myocardial infarction (0.18% versus 0.23% per year, *P* < 0.0001) [23]. The net effect on stroke was not significant (0.20% versus 0.21% per year, *P* = 0.4: haemorrhagic stroke 0.04% versus 0.03%, *P* = 0.05; other strokes 0.16% versus 0.18% per year, *P* = 0.08). Vascular mortality did not differ significantly (0.19% versus 0.19% per year, *P* = 0.7). Aspirin use increased major gastrointestinal and extracranial bleeds (0.10% versus 0.07% per year, *P* < 0.0001), and the main risk factors for coronary disease were also risk factors for bleeding.

To better understand the impact of sex on the response to aspirin, Berger and colleagues conducted a meta-analysis on the sex-specific benefits of aspirin in 51342 women and 44114 men enrolled in the 6 previous prevention trials [50]. The results demonstrate that aspirin therapy is associated with a significant reduction in the risk of cardiovascular events in both sexes. However, the specific types of benefit differ in important ways between women and men. Aspirin use in women was associated with statistically significant reductions in cardiovascular events (odds ratio [OR], 0.88 [CI, 0.79 to 0.99]) and ischemic strokes (OR, 0.76 [CI, 0.63 to 0.93]); no statistically significant benefit was found in the reduction of myocardial infarctions or cardiovascular mortality. In men, aspirin use was associated with a statistically significant reduction in cardiovascular events (OR, 0.86 [CI, 0.78 to 0.94]) and myocardial infarctions (OR, 0.68 [CI, 0.54 to 0.86]); no statistically significant benefit was found in the reduction of ischemic strokes or cardiovascular mortality. Total mortality was not significantly reduced by aspirin use in men or women. 

In summary, consistent evidence from randomized clinical trials indicates that aspirin use reduces the risk for CVD events in adults without a history of CVD. For primary prevention of cardiovascular disease, aspirin therapy significantly reduced the risk of the composite of cardiovascular events primarily by reducing the risk of ischemic stroke with no significant effect on the risk of MI in women and predominantly by reducing the risk of MI with no significant effect on the risk of stroke in men.

## 6. Adverse Effects

Aspirin prevents thrombotic events by inhibiting prostaglandin synthesis, which also leads to adverse side effects, mainly including upper-gastrointestinal (GI) toxicity, extracranial and intracranial haemorrhage [51–53].

Aspirin-induced GI toxicity detected in randomized clinical trials, including nausea, heartburn, and epigastric pain, appears to be dose related in the range of 30 to 1,300 mg/d. The principle mechanism is due to the inhibition of COX-1-dependent prostaglandin E2 (PGE2) synthesis by aspirin, while PGE2 inhibits acid secretion in gastric mucosa and increases mucous formation. Buffered and enteric-coated aspirin preparations developed to attenuate local gastric erosion and minimize this side effect [51].

The overall risk of major extracranial and intracranial hemorrhage associated with antiplatelet drugs is difficult to assess in individual trials because their incidence is low [52, 53]. In the overview of the ATT Collaboration [17], the overall proportional increase in risk of a major extracranial hemorrhage with aspirin therapy was about one-half (odds ratio [OR], 1.6; 95% CI, 1.4 to 1.8). After allowing for noncompliance in the trials, they are compatible with the 2- to 2.5-fold excess observed in case-control studies. The overall absolute excess of intracranial hemorrhage due to aspirin therapy was <1 per 1000 patients per year in high-risk trials, with somewhat higher risks in patients with cerebrovascular disease.

Moreover, chronic large dose of aspirin use may reduce renal blood flow and glomerular filtration and impair renal function due to the inhibition of COX-2-dependent PGI2, which support renal perfusion, diminish vascular resistance, and facilitate natriuresis [54]. This side effect often occurs at high aspirin doses and most frequently in elderly patients and those with established renal disease.

Furthermore, high-dose aspirin may also attenuate the benefit of angiotensin-converting enzyme (ACE) inhibitors in hypertensive and congestive heart failure patients because aspirin may attenuate the synthesis of PGE3 and PGI2, which is promoted by ACE inhibitors [55–57].

In summary, aspirin is effective for the prevention of thrombosis because of the inhibition of TXA2-dependent platelet function, which is also associated with excess bleeding. Assessing the net effect requires an estimation of the absolute thrombotic versus hemorrhagic risk of the individual patient. 

## 7. Aspirin Resistance

Aspirin resistance has been used to describe the inability of aspirin to protect individuals from thrombotic complications, cause a prolongation of the bleeding time, reduce TXA2 production, or produce typical effect in vitro tests of platelet function [58, 59]. However, a standard, clear, and distinct definition of aspirin resistance has not been established yet. 

The rate of aspirin resistance is widely variable, ranging from 5 to 60% of the population affected by cardiovascular and cerebrovascular diseases in different studies [58–60]. It is difficult to know the exact prevalence of aspirin resistance from these studies because of variabilities in definitions for aspirin resistance, variabilities in testing and measurement between studies, small sample size of the studies, and different populations used in the studies. Many laboratory tests are currently used to investigate platelet activity and platelet response to aspirin, such as measurements of thromboxane biosynthesis, platelet aggregation, and platelet activation, bleeding time.

The potential mechanisms of aspirin resistance include enhanced platelet turnover, genetic polymorphisms of COX-1 and other genes involved in thromboxane biosynthesis, upregulation of nonplatelet sources of thromboxane biosynthesis, and the interactions of other drugs [58, 61, 62].

Because of a series of adverse cardiovascular events associated with aspirin resistance, once aspirin resistance is confirmed by laboratory measures, recommendations for alteration of therapy (dose change or additional antiplatelet agent) and followup are needed for meaningful clinical outcomes.

## 8. Conclusions

Aspirin remains the cornerstone of antiplatelet therapy in patients with cardiovascular disease. It decreases mortality and recurrence of cardiovascular events when used as acute therapy following acute coronary syndrome, thrombotic stroke, and Kawasaki's disease. It is also of proven benefit in secondary prevention among a wide range of patients, including those with acute coronary syndrome, stable angina, revascularization, stroke, TIA, and atrial fibrillation. In primary prevention, aspirin therapy appears to reduce the risk for CVD events in adults without a history of CVD with sex specific benefits. Aspirin may be considered for patients with a high risk of future CVD, but the benefits must be weighed against the possibility of side effects. The concept of resistance to aspirin is still an emerging and important clinical question requiring further study.
